# Adopting a Theophylline-Responsive Riboswitch for Flexible Regulation and Understanding of Glycogen Metabolism in *Synechococcus elongatus* PCC7942

**DOI:** 10.3389/fmicb.2019.00551

**Published:** 2019-03-21

**Authors:** Xintong Chi, Shanshan Zhang, Huili Sun, Yangkai Duan, Cuncun Qiao, Guodong Luan, Xuefeng Lu

**Affiliations:** ^1^Key Laboratory of Biofuels, Qingdao Institute of Bioenergy and Bioprocess Technology, Chinese Academy of Sciences, Qingdao, China; ^2^Shandong Provincial Key Laboratory of Synthetic Biology, Qingdao Institute of Bioenergy and Bioprocess Technology, Chinese Academy of Sciences, Qingdao, China; ^3^College of Life Science and Technology, Central South University of Forestry and Technology, Changsha, China; ^4^College of Life Science, University of Chinese Academy of Sciences, Beijing, China; ^5^Shandong Provincial Key Laboratory of Energy Genetics, Qingdao Institute of Bioenergy and Bioprocess Technology, Chinese Academy of Sciences, Qingdao, China; ^6^Dalian National Laboratory for Clean Energy, Dalian, China; ^7^Laboratory for Marine Biology and Biotechnology, Qingdao National Laboratory for Marine Science and Technology, Qingdao, China

**Keywords:** cyanobacteria, glycogen, GlgC, riboswitch, photosynthesis, stress tolerance

## Abstract

Cyanobacteria are supposed to be promising photosynthetic microbial platforms that recycle carbon dioxide driven into biomass and bioproducts by solar energy. Glycogen synthesis serves as an essential natural carbon sink mechanism, storing a large portion of energy and organic carbon source of photosynthesis. Engineering glycogen metabolism to harness and rewire carbon flow is an important strategy to optimize efficacy of cyanobacteria platforms. ADP-glucose pyrophosphorylase (GlgC) catalyzes the rate-limiting step for glycogen synthesis. However, knockout of *glgC* fails to promote cell growth or photosynthetic production in cyanobacteria, on the contrary, *glgC* deficiency impairs cellular fitness and robustness. In this work, we adopted a theophylline-responsive riboswitch to engineer and control *glgC* expression in *Synechococcus elongatus* PCC7942 and achieved flexible regulation of intracellular GlgC abundance and glycogen storage. With this approach, glycogen synthesis and glycogen contents in PCC7942 cells could be regulated in a range from about 40 to 300% of wild type levels. In addition, the results supported a positive role of glycogen metabolism in cyanobacteria cellular robustness. When glycogen storage was reduced, cellular physiology and growth under standard conditions was not impaired, while cellular tolerance toward environmental stresses was weakened. While when glycogen synthesis was enhanced, cells of PCC7942 displayed optimized cellular robustness. Our findings emphasize the significance of glycogen metabolism for cyanobacterial physiology and the importance of flexible approaches for engineering and understanding cellular physiology and metabolism.

## Introduction

Cyanobacteria are photoautotrophic prokaryotes, that are widespread in diverse ecosystems, including the ocean, fresh water, and terrestrial environments (Waterbury et al., 1979). They evolved oxygenic photosynthesis, an efficient system converting solar energy and CO_2_ into organic compounds (Hohmann-Marriott and Blankenship, 2011). Currently, cyanobacteria produces 10–20% of the organic carbon on Earth, playing an essential role in global carbon and nitrogen cycles (Flombaum et al., 2013; Rousseaux and Gregg, 2014). A large portion of the photosynthetically assimilated carbon source, which excesses the normal requirements of cell growth and metabolism in cyanobacteria, is stored in the form of glycogen, a high molecular branched α-polyglucan (Ball and Morell, 2003; Nakamura et al., 2005). Its pool can account for up to 50% of the total cellular biomass under specific environmental conditions (Aikawa et al., 2014; Song et al., 2016). During the night or in the absence of a carbon source, carbon and energy stored in the glycogen is mobilized and used for the central metabolism (Stal and Moezelaar, 1997; Guerra et al., 2013). In addition, there are hints that glycogen metabolism protects cyanobacteria against unfavorable environmental conditions (Suzuki et al., 2010; Grundel et al., 2012; Hickman et al., 2013).

During the last decade, cyanobacteria were increasingly recognized as efficient photosynthetic platforms that can used to recycle CO_2_ into biomass and bioproducts, using solar energy. The use of cyanobacteria for biotechnological application is also supported by their simple structure, rapid growth, and existing tools for genetic manipulation (Angermayr et al., 2009; Lu, 2010). To improve the efficacy of photosynthetic platforms, a promising strategy would be engineering glycogen metabolism in cyanobacteria for better rewiring and to harness the organic carbon output from the Calven-Benson-Bassham cycle (Carrieri et al., 2012).

**Figure 1:**
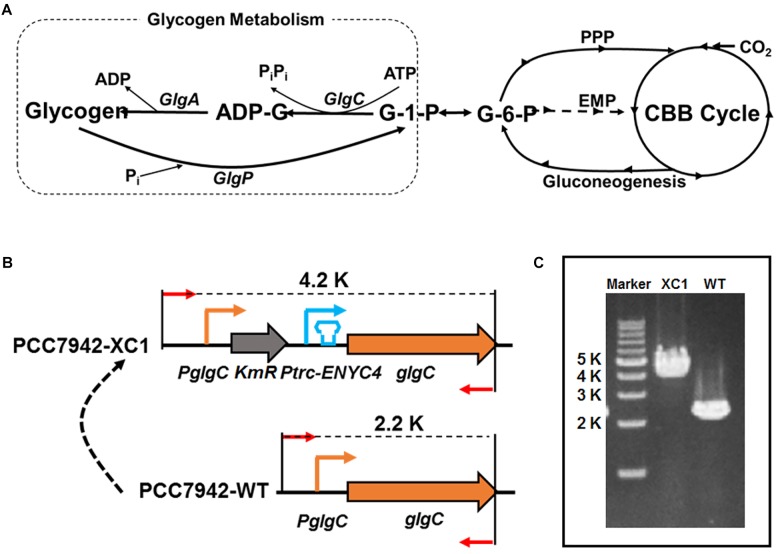
Development of a theophylline-responsive *glgC*-expression system in *Synechococcus elongatus* PCC7942. **(A)** Schematic representation of glycogen metabolism in cyanobacteria. GlgP, glycogen phosphorylase; GlgC, glucose-1-phosphate adenylyltransferase or ADP-glucose pyrophosphorylase; GlgA, glycogen synthase; G-1-P, glucose-1-phosphate; G-6-P, glucose-6-phosphate; ADP-G, ADP-glucose; PPP, pentose phosphate pathway; EMP, Embden-Meyerhof-Parnas pathway, glycolytic pathway; CBB cycle, Calvin-Benson-Bassham cycle; Pi, inorganic phosphate; PiPi, pyrophosphate. **(B)** Construction strategy of a theophylline-responsive riboswitch control system on *glgC* expression in PCC7942. *KmR*, kanamycin resistance gene; *PglgC*, native promoter sequence of glgC. **(C)** Genotype identification of the PCC7942-XC1 and PCC7942-WT by PCR.

As shown in Figure 1A, cyanobacterial glycogen synthesis starts from the precursor glucose 1-phosphate (G-1-P), which is stepwisely converted into glycogen by the action of ADP-glucose pyrophosphorylase (GlgC, also termed glucose-1-phosphate adenylyltransferase) catalyzing ADP-glucose (ADP-G) formation, glycogen synthase (GlgA) incorporating glucose monomers into the growing 1-4 α-linked glucose polymer, and branching enzyme introducing 1-6 branches connecting the linear polyglucose chains. The activity of GlgC represents the rate-limiting step and controls the glycogen accumulation process (Ball and Morell, 2003; Grundel et al., 2012). Thus, many attempts have been published to knock-out the *glgC* gene in cyanobacteria, to eliminate glycogen accumulation in diverse cyanobacterial strains. However, impaired glycogen accumulation severely affected cell physiology, including reduced photosynthesis, growth, respiration, and cellular robustness in facing environmental stresses (Miao et al., 2003; Suzuki et al., 2010; Carrieri et al., 2012; Grundel et al., 2012; Guerra et al., 2013; Hickman et al., 2013). In many cases, deficient glycogen synthesis decreased rather than increased the productivity of heterologous pathways in engineered cyanobacterial strains (Davies et al., 2014; Jacobsen and Frigaard, 2014; Li et al., 2014; van der Woude et al., 2014; Work et al., 2015). In summary, when the glycogen synthesis pathway was rigidly and completely blocked (through deletion of *glgC*), carbon flow can neither be effectively rewired toward cell growth nor to desired non-natural metabolites, indicating that more efforts and approaches are still required to understand and harness cyanobacterial glycogen metabolism.

In this work, we aimed to engineer and decipher the physiological function of cyanobacteria glycogen metabolism in a flexible and regulatable mode. To this end, a theophylline-responsive riboswitch was adopted to control the expression of *glgC* in *Synechococcus elongatus* PCC7942 (hereafter referred to as PCC7942), permitting controllable down- and up-regulated glycogen synthesis and storage in the same system. Based on this flexible approach, the influence of glycogen metabolism on cyanobacteria cellular physiology was explored. The data we obtained in this work supported the positive role of glycogen synthesis and contents on cyanobacteria cellular fitness and robustness toward environmental stresses.

## Results and Discussion

### Construction of a Theophylline-Responsive *glgC* Expression System in PCC7942

GlgC catalyzes the rate-limiting step of glycogen synthesis (Figure 1A) and is generally supposed to maintain control over all glycogen metabolism activities in cyanobacteria (Ball and Morell, 2003; Grundel et al., 2012). Thus, we selected *glgC* (*Synpcc7942_0603*) in PCC7942 as the target to engineer and regulate. To develop artificial control over the *glgC* expression, we adopted a synthetic theophylline-responsive riboswitch system (*ENYC4*) (Nakahira et al., 2013) to achieve strict regulation of intracellular protein abundance. As shown in Figure 1B, a cassette containing kanamycin-resistance gene (*KmR*), a *Ptrc* promoter and a theophylline-responsive riboswitch region (*Ptrc-ENYC4*) was inserted into the chromosome of PCC7942 between the *glgC* CDS region and the native promoter sequence. The integration was confirmed by PCR (Figure 1C) and DNA sequencing. The PCC7942 mutant carrying the *KmR*-*Ptrc-ENYC4-glgC* cassette on the chromosome was termed as PCC7942-XC1 (XC1), while the wild type strain of PCC7942 was termed as PCC7942-WT (WT) as a control.

### Theophylline-Dose Regulated *glgC* Expression and Glycogen Storage in PCC7942-XC1

**Figure 2:**
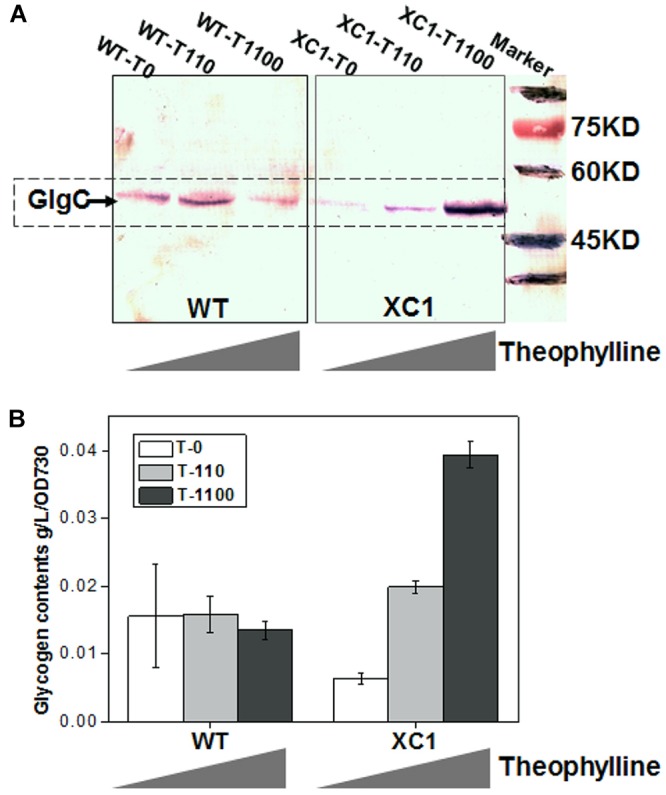
Theophylline-dose regulated GlgC abundances and glycogen storage in PCC7942-XC1. **(A)** Western blot assay for GlgC concentrations in WT strain and XC1 strain under serial theophylline concentrations (T0, no theophylline addition; T110, 110 μM theophylline; T1100, 1100 μM theophylline). In PCC7942, *glgC* gene encodes a protein with MW of about 48 kDa, consistent with the band locations marked in the PVDF membrane. **(B)** Glycogen contents in WT strain and XC1 strain under serial theophylline concentrations. The data shown are averages of at least three independent biological repeats, and standard deviation bars are also shown.

To evaluate the effects of theophylline-responsive riboswitch on controlling *glgC* expression, three different concentrations (0, 110, 1100 μM) of theophylline were supplemented into BG11 culture medium of PCC7942-WT and PCC7942-XC1, and the GlgC abundances in the two strains were determined by western-blot. As shown in Figure 2A (XC1 region), GlgC concentrations in PCC7942-XC1 were correlated with the theophylline concentrations. GlgC abundance in XC1 strain cells without theophylline addition (XC1-T0) was significantly reduced, compared to that of the PCC7942-WT (WT-T0). When theophylline concentrations were supplemented at 1100 μM, the GlgC concentrations (XC1-T1100) were much higher than that of the control (WT-T0, T110, T1100), indicating that expression of *glgC* was successfully controlled by the regulation of theophylline-dose.

Glycogen storage in XC1 strain could also be successfully regulated by the theophylline-dose. As shown in Figure 2B and Supplementary Figure S1, when no theophylline was supplemented, glycogen contents in the cellular biomass of XC1 was approximately 40% of that in the wild type control (6.4 mg/L/OD730 for XC1-T0 and 15.6 mg/L/OD730 for WT-T0), which is consistent with the lowered GlgC abundances in XC1-T0. When theophylline concentrations were increased to 110 μM, the glycogen contents in XC1 (19.8 mg/L/OD730) was about 25% higher than that of the wild type control (15.8 mg/L/OD730 for WT-T110). When the theophylline concentrations were further increased to 1100 μM, glycogen contents in XC1 (39.4 mg/L/OD730 for XC1-T1100) were increased threefold higher than the wild type control in the same conditions (13.9 mg/L/OD730 for WT-T1100). In summary, glycogen synthesis and storage in PCC7942-WT cells were not significantly altered in response to theophylline addition, while the glycogen contents of XC1 cells could be regulated in a range from about 40 to 300% of the wild type level by theophylline-dose regulation.

It has previously been reported that glycogen storage in cyanobacteria could be down-regulated by some recently existing interfering approaches. In *Synechocystis* sp. PCC6803, Yao et al. (2016) adopted a CRISPR interference (CRISPRi) tool to knock down the expression of *glgC*. Through an inducible expression of the *dCAS9* element, the transcription of *glgC* was decreased by 90%, while the glycogen contents were reduced by 70% compared to the same in the wild type control under nitrogen deprivation conditions. Sun and colleagues developed small RNA regulatory tools based on paired termini RNAs and an exogenous MicC scaffold combined with the Hfq chaperone (Hfq-MicC). The sRNA regulatory system repressed expression of *glgC* by over 90%, and resulted in a 75% decreased glycogen content (Sun et al., 2018). In PCC7942, Huang and colleagues also adopted the CRISPRi approach for down-regulated expression of *glgC*, and successfully reduced glycogen contents by 75%. In general, intracellular glycogen contents in cyanobacteria could be decreased by 70 to 90% with the CRISPRi strategy and a small RNA regulatory tool, permitting controllable repression of *glgC* and reduction of glycogen contents. In comparison, the riboswitch approach adopted in this work enabled a bidirectional regulation of glycogen synthesis and storage, meaning both up-regulation and down-regulation could be achieved in a single system. Through dose-regulation of the signal molecules, physiological characteristics of the same cyanobacteria strain containing reduced or over-accumulated glycogen could be directly evaluated and compared, which would benefit the comprehensive understanding of glycogen metabolism effects on cyanobacteria cellular fitness and robustness.

### Reduced Glycogen Synthesis Did Not Impair Cell Growth of PCC7942

**Figure 3:**
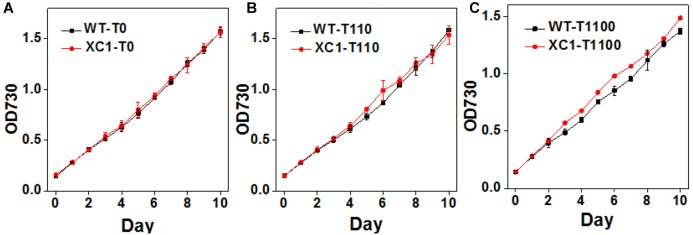
Growth assays of PCC7942-WT and PCC7942-XC1 under induction of gradually increasing theophylline concentrations. **(A)** No theophylline was added; **(B)** 110 μM theophylline was added; **(C)** 1100 μM theophylline was added. Optical densities under 730 nm (OD730) were used to calculate cell concentrations. The data utilized are averages of measurements from at least three independent biological repeats, and the standard deviations bars are shown.

Previously, it has been generally reported that abolished glycogen synthesis and storage would lead to significant retarded cell growths in diverse cyanobacteria strains, including *Synechocystis* sp. PCC6803 (Miao et al., 2003), *Synechococcus elongatus* PCC7942 (Suzuki et al., 2010), and *Synechococcus* sp. PCC7002 (Guerra et al., 2013). Thus, cell growths of PCC7942-XC1 were first characterized under gradually increased theophylline concentrations. As shown in Figure 3A, when no theophylline was added to induce the synthesis of glycogen, cell growths of PCC7942-XC1 were indistinguishable from that of PCC7942-WT. After 10 days of cultivation, no significant difference was observed on cell densities of the two strains with different glycogen contents (Figure 2B). When the theophylline concentration was increased to 110 μM, cell growths of the two strains were still maintained on a similar level (Figure 3B). While, as shown in Figure 3C, when the theophylline significantly positively-relates to 1100 μM, slight growth advantages of XC1 cell growth were observed over that of PCC7942-WT, indicating that increased glycogen contents might compensate the inhibitory effects on PCC7942 growth from high concentrations of theophylline.

**Figure 4:**
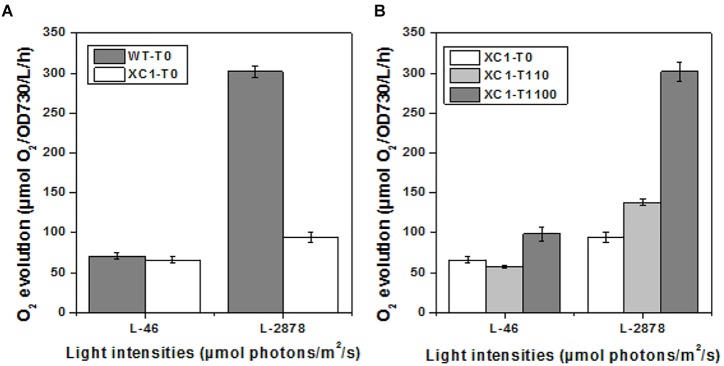
Oxygen evolution of PCC7942-WT and PCC7942-XC1 cells under different illumination. L-46 represented light intensities of 46 μmol/m^2^/s; L-2878 represented light intensities of 2878 μmol/m^2^/s. The data shown are averages of at least three independent measurements, and standard deviation bars are also shown. **(A)** PCC7942-WT and PCC7942-XC1 under 0 μM theophylline; **(B)** PCC7942-XC1 under induction of 0 μM, 110 μM, 1100 μM theophylline, respectively.

To further explore the influence of regulated glycogen synthesis and storage on cyanobacterial cellular physiology, we assayed oxygen evolution rates in PCC7942-XC1 and the wild type control. As shown in Figure 4A, when light intensities were set at a low level (46 μmol photons/m^2^/s), which was utilized in cultivation, the oxygen evolution rate in XC1-T0 was similar to that of the wild type control (WT-T0). And that phenomenon was consistent with similar growth profiles between the XC1 strain and the wild type control when no theophylline was supplemented.

Additionally, when high light intensities (2878 μmol photons/m^2^/s) were provided, oxygen evolution activities in the wild type strain of PCC7942 were significantly increased, while the increase in XC1 was much more limited, indicating that the capacities to tolerate and utilize strong illuminations of PCC7942 cells were impaired by the reduced glycogen synthesis and storage. The results were consistent with a previous observation in a *glgC*-null mutant of PCC7942 (Suzuki et al., 2010). A hypothesis was proposed that organic carbon output rates exceeded the consuming rates of cellular growth in cyanobacteria, while excessed carbon and energy would prevent higher carbon fixation rates in photosynthesis (Oliver and Atsumi, 2015). Glycogen storage serves as a natural carbon sink mechanism and can remove restrictions on photosynthesis. In addition, strong illuminations would also cause physiological impairments on cyanobacteria. Glycogen metabolism also plays an important role in maintaining cellular robustness when facing high light stress followed by oxygenic stress (Suzuki et al., 2010; Grundel et al., 2012). Thus, the weakened cellular robustness caused by reduced glycogen metabolism in XC1 (T0) might also contribute to the inhibited photosynthesis activities compared to that of the PCC7942-WT cells. The inhibited oxygen evolution activities under high light intensities could be relieved by the addition of theophylline and increased glycogen synthesis in XC1 (Figure 4B), confirming an important role of glycogen synthesis and storage in tolerating strong illumination stress and buffering carbon and energy excess from photosynthesis.

As has been generally discovered and accepted in previous research, completely abolishing glycogen synthesis and storage, would inhibit cyanobacterial cellular growths and photosynthesis activities, even when cultivated under normal conditions with continuous illuminations (Miao et al., 2003; Suzuki et al., 2010; Grundel et al., 2012). However, our results provides evidence showing that when glycogen synthesis and storage is reduced rather than completely removed, the defects on cell growth and cellular physiology under normal conditions can be avoided. In addition, the important role of glycogen synthesis and storage for buffering the excess of light energy input was also confirmed in this work.

### Glycogen Synthesis Promoted Cellular Tolerance to Environmental Stresses

**Figure 5:**
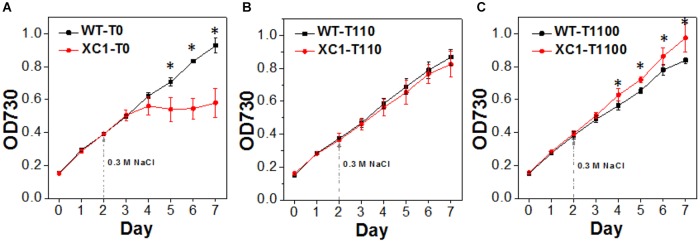
Growths of PCC7942-WT and PCC7942-XC1 facing NaCl stress under theophylline concentrations of **(A)** 0 μM, **(B)** 110 μM, and **(C)** 1100 μM. 0.3 M NaCl was supplemented into the culture medium in Day 2 as the arrow pointed. The OD730 data utilized are averages of measurements from at least three independent biological repeats, and standard deviation bars are also shown. Asterisksg49 indicate significant differences between the data of WT and XC1 (Student’s *t*-test, *P* < 0.05).

Glycogen metabolism has been proposed to play an important role for cyanobacteria, to resist multiple environmental stresses (Preiss, 1984; Suzuki et al., 2010; Grundel et al., 2012; Hickman et al., 2013). Thus, we also evaluated the stress tolerances dynamics of the XC1 strains with theophylline-concentration regulated glycogen synthesis and storage. Previously it has been reported that knockout of the *glgC* gene in PCC6803 (Miao et al., 2003), PCC7942 (Suzuki et al., 2010), and PCC7002 (Guerra et al., 2013) resulted in decreased cellular tolerance to NaCl stress. In this work, we observed similar results. As shown in Figure 5A, when 0.3 M NaCl was added to stressed PCC7942 cells, the XC1 strain without theophylline induction (XC1-T0) showed significant reduced cell growth compared with the WT-T0 control. The cell growth of XC1-T0 under 0.3 M NaCl stress continued for less than 2 days and entered into a stationary phase (OD730 ∼ 0.6), while that of WT-T0 continued for over 5 days, OD730 reaching up to 0.9. When theophylline was supplemented to induce glycogen synthesis and accumulations, growth capacities of the XC1 strain facing NaCl stress were gradually recovered and enhanced to levels even surpassing those of the WT controls (Figure 5B,C). While as for PCC7942-WT, cell growth was slightly retarded by increasing concentrations and toxicities of theophylline (Figure 5B,C). In our previous research, we reported that, as the main compatible solute of PCC7942 to resist osmotic stress, synthesis of sucrose is closely linked to glycogen accumulations (Qiao et al., 2018), which might be a reason for the advantages of the XC1 strain with increased glycogen synthesis.

**Figure 6:**
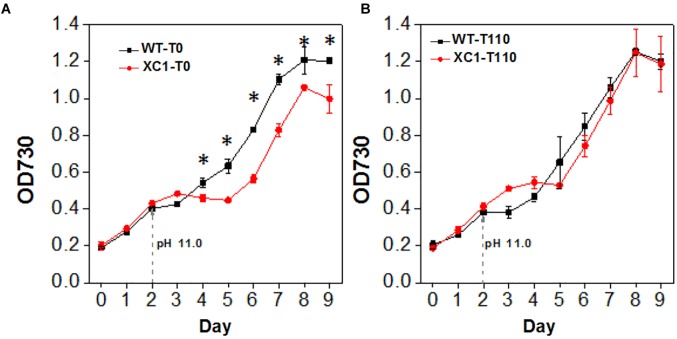
Growth of PCC7942-WT and PCC7942-XC1 facing high pH stress under theophylline concentrations of **(A)** 0 μM, **(B)** 110 μM. To adjust pH values of the culture medium to 11.0, NaOH would be added in day 2 as the arrow pointed. The OD730 data utilized are averages of measurements from at least three independent biological repeats, and standard deviation bars are also shown. Asterisks’ indicate significant differences between the data of WT and XC1 (Student’s *t*-test, *P* < 0.05).

We also explored the effects of glycogen synthesis and contents on cellular tolerance to high pH stress. Similar to the growth pattern in high salt conditions, when the pH value of the culture medium was increased to 11, growth of XC1 (without theophylline induction, XC1-T0) was significantly decreased (Figure 6A). Addition of 110 μM theophylline, under which the glycogen content of XC1 cells was comparable with that of the wild type control, relieved the growth defects in XC1 (Figure 6B), indicating that glycogen synthesis and storage is an important approach of cyanobacteria to resist alkaline stress.

Stress-responsive cellular activities required additional supply of energy and materials to maintain homeostasis. Glycogen storage served as an essential substrate for respiration, thus fulfilling emergent requirements (Guerra et al., 2013). It has been generally accepted that deficiency of glycogen synthesis would result in impaired tolerance to abiotic stresses (Preiss, 1984; Suzuki et al., 2010; Grundel et al., 2012; Hickman et al., 2013). In this work, our results further provided solid data supporting the positive role of glycogen metabolism in cellular robustness.

## Conclusion

Glycogen synthesis is an essential natural carbon sink mechanism found in cyanobacteria, that stores carbon and energy derived from photosynthesis. Engineering the glycogen metabolism of cyanobacteria is required for rewiring the intracellular carbon flow and to optimize the efficacy of the photosynthetic platform. It has previously been reported that the *glgC*-knock-out strategy impaired cellular fitness, therefore, in this work we adopted a theophylline-responsive riboswitch system to control *glgC* expression in PCC7942. Through the regulation of theophylline concentrations, GlgC abundance and glycogen storage could be reversibly regulated in a range from 40 to 300% of the wild type level. With this bidirectional regulation approach, a more flexible understanding of the glycogen metabolism effects on cellular fitness and robustness in cyanobacteria could be obtained. When glycogen storage was reduced, the growth of PCC7942 under normal conditions was maintained, while tolerance to NaCl and high pH stress was inhibited. When glycogen storage was elevated, improved stress-tolerances were endowed in the engineered strain. Our results further highlight the significance of flexible and dynamic regulation tools for engineering and understanding cellular physiological and metabolic activities.

## Materials and Methods

### Chemicals and Reagents

Unless noted otherwise, all reagents were purchased from Sigma-Aldrich (United States). *Taq* DNA polymerase and all restriction enzymes were purchased from Fermentas (Canada) or Takara (Japan). The kits used for molecular cloning were from Omega (United States) or Takara (Japan). Oligonucleotides were synthesized and DNA sequencing was performed by Genewiz (Suzhou, China).

### Strain Construction and Cultivation

*Escherichia coli* DH5α was used for plasmids construction in this work. To achieve a theophylline-dependent riboswitch controlled *glgC* expression, a previously developed plasmid pDY123 (Qiao et al., 2018) was used to transform the wild type strain of *Synechococcus elongatus* PCC7942 (PCC7942-WT). For construction of pDY123, the P*trc*-ENYC4 sequence was first synthesized according to the reported nucleotide sequence (Nakahira et al., 2013), which contains the *trc* promoter (P*trc*) and theophylline responsive riboswitch sequence (ENYC4), and then the sequence was inserted into the cloning vector pMD18-T simple (TaKaRa, Dalian, China). Second, the P*trc*-ENYC4-glgC fragments were obtained by fusion PCR (using primers E-F/glgC-R), which used the P*trc*-ENYC4 fragment (amplified by E-F/E-glgC-R) and *glgC* open reading frame (ORF) fragment (amplified by glgC-F/glgC-R). Additionally, the resistance fragment glgCUP-KanR was obtained by merging the *glgC* upstream fragment with the Kanamycin resistant fragment from pXT212 (Tan et al., 2013). Then, pDY121 and pDY122 were constructed by inserting glgCUP-KanR and P*trc*-ENYC4–*glgC* into the vector (pJET1.2/blunt) respectively. Finally, glgCUP-KanR was digested by SpeI from pDY121 and inserted into the upstream site (XbaI) of the P*trc*-ENYC4-*glgC* fragment of pDY122, to construct the target plasmid pDY123. Primer information utilized for construction of pDY123 is listed in Supplementary Table S1. After transformation of pDY123 to PCC7942, kanamycin-resistant transformants were obtained after 7–10 days of selective cultivation on BG11 agar plates. The genotypes of the transformants were verified by PCR and DNA-sequencing. The final obtained mutant strain was named XC1 (PCC7942-XC1).

For growth assays, *Synechococcus* strains were cultivated in BG11 medium added with 10 mM TES-NaOH with (for PCC7942-XC1) or without (for PCC7942-WT) 20 μg/ml kanamycin. Cells were cultivated at 30°C in an incubator with 150 rpm under moderate intensity white-light illumination (30–50 μmol photons/m^2^/s). Cells of PCC7942-WT or PCC7942-XC1 were inoculated into the culture medium to the initial OD730 of about 0.2 and then during the cultivation process the growths were calculated by measuring OD730. As shown in Supplementary Figure S2, dry cell weights of WT and XC1 cells under different theophylline concentrations were similar when the OD730 was the same. To evaluate the effects of theophylline-induced glycogen synthesis and storage on cellular growth and physiology, concentrations of theophylline were supplemented into the culture medium as simultaneously required with the inoculation.

For the stress-resistance assay of the *Synechococcus* strains, appropriate amounts of NaCl (final NaCl concentration of 0.3 M) or NaOH (final pH 11) were added to the culture medium as required after 2 days of cultivation when the OD730 reached about 0.4.

For growth assays and comparisons between PCC7942-WT and PCC7942-XC1 under different conditions, at least three independent biological repeats were performed for each experiments batch and the separate-day experiments were also performed to confirm trends.

### Western-Blot for GlgC Abundances

As previously described (Qiao et al., 2018), a 121-amino-acid fragment of GlgC from PCC7942 (positions 230 to 350) was utilized to prepare rabbit-sourced antibodies against PCC7942 GlgC (Hua’an Bio, Hangzhou, China). *Synechococcus* cells were harvested by centrifugation at 4°C. The resuspended cells (in 50 mM Tris-HCl buffer, pH 8.0) were disrupted with 100 μm glass beads (Sigma) with ice bath. After removing the cell debris and glass beads by 4°C centrifugation, the supernatants were collected. Protein concentrations of the cell-free extracts were also measured with Bradford method. The protein samples were analyzed on 12% SDS-PAGE with a standard procedure (the gel picture was supplemented as Supplementary Figure S1) and blotted to PVDF membrane, sealed in 5% non-fat milk-TBST buffer (TBS added with 0.05% Tween-20) at 4°C overnight. First, the membrane was incubated with polyclonal antibodies to GlgC (1:1000) for 3 h and washed three times with TBST (15 min each time). Second, the membrane was incubated with an alkaline phosphatase-linked secondary antibody (goat anti rabbit, Invitrogen, Shanghai, China) for 1 h and washed three times with TBST (15 min each time). Finally, the membrane was developed with BCIP/NBT (Sigma).

### Glycogen Contents Calculation

Glycogen contents of *Synechococcus* strains were measured as previously introduced with modifications (Grundel et al., 2012). *Synechococcus* cells cultivated in BG11 broth under normal condition were collected at day 6. The cells were centrifuged and washed twice with distilled water. The cells were finally resuspended with 400 μl 30% KOH and heated at 95°C for 2 h. Chilled ethanol was added to the lysate solution to the final concentration of 70% (V/V). The mixture was cooled on ice for 4 h, and then centrifuged (14000 *g*, 15 min, and 4°C). The pellet was washed with 70% ethanol (V/V) and 98% ethanol (V/V) successively, and then dried by vacuum centrifugation. The glycogen finally obtained was suspended in 500 μl 100 mM sodium acetate and digested by 2 mg amyloglucosidase (Novozymes). Glucose contents generated in the glycogen solution was determined with an SBA-40c biosensor analyzer (Shandong Academy of Sciences, China) and used for calculation of the glycogen contents.

### Photosynthetic Activity Analysis

Oxygen evolution rates were calculated with Clark-type oxygen electrode at 30°C with light intensity of 46 or 2878 μmol photons/m^2^/s. PCC7942 samples were collected from BG11 culture broth supplemented with different theophylline concentrations (as introduced in the “Strain construction and cultivation” part) at day 6 under normal conditions, when the OD730 was about 0.8–1.0.

## Data Availability

All datasets generated for this study are included in the manuscript and/or the Supplementary Files.

## Author Contributions

XC and SZ performed the experiments and participated in manuscript preparation. HS, YD, and CQ participated in the research. GL and XL designed and directed the research and wrote and revised the manuscript. All of the authors have read the final manuscript and agreed to submit it to Frontiers in Microbiology.

## Conflict of Interest Statement

The authors declare that the research was conducted in the absence of any commercial or financial relationships that could be construed as a potential conflict of interest.
