# Supplementary material for: Adopting a Theophylline-Responsive Riboswitch for Flexible Regulation and Understanding of Glycogen Metabolism in Synechococcus elongatus PCC7942
Source: Front Microbiol. 2019 Mar 21;10:551. doi: 10.3389/fmicb.2019.00551 (PMC6437101; doi:10.3389/fmicb.2019.00551)
Supplement: Supplementary file 1 [file Table_1.DOCX]

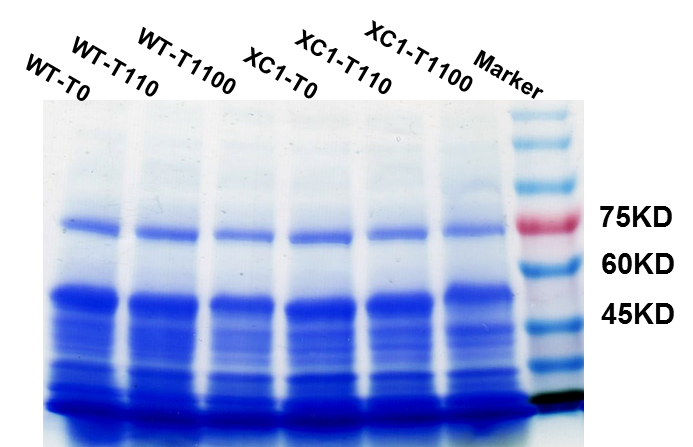


**Figure S1. SDS-Page assays of the theophylline-dose regulated protein patterns in crude cell extracts of PCC7942-XC1 and PCC7942-WT under serial theophylline concentrations (T0, no theophylline addition; T110, 110 μM theophylline; T1100, 1100 μM theophylline). The same cell extracts were used for western blot assays of GlgC abundances.**

**Figure S2. Dry cell weights assays of PCC7942-WT and PCC7942-XC1 under gradient concentrations of theophylline. The data are presented as means from three independent replicates. For dry cell weight measurements, 20 ml culture broth of PCC7942-WT or PCC7942-XC1 was concentrated, and then washed and resuspended with 50 mM Tris-HCl buffer. The cells would finally be collected on nitrocellulose membrane and dried completely for biomass contents measurement.**

**Table S1. Primers information utilized for construction of plasmid of pDY123 and the sequence of P*trc*-ENYC4**

| Primers | Sequence |
| --- | --- |
| E-F | AAATATTCTGAAATGAGCTGTTGAC |
| E-glgC-R | ATGATCGCCAGCACGTTTTTCACCTTGTTGCCTCCTTAGCAGGGT |
| glgC-F | ACCCTGCTAAGGAGGCAACAAGGTGAAAAACGTGCTGGCGATCAT |
| glgC-R | TTAGATCACCGTGTTGTCGGG |
| rpe-F | ACTAGTCAGCGTTCACCTCAAGCAACT |
| rpe-km-R | AGACGTGTAATGCTGCAATCTAATCAATCTCCCCCAAGTCAAG |
| rpe-km-F | CTTGACTTGGGGGAGATTGATTAGATTGCAGCATTACACGTCT |
| km-R | ACTAGTGTGACACAGGAACACTTAACGGC |
| *Ptrc-ENYC4 sequence*：  AAATATTCTGAAATGAGCTGTTGACAATTAATCATCCGGCTCGTATAATGTGTGGAATTGTGAGCGGATAACAATTTCATACGCTCACAATTGGTACCGGTGATACCAGCATCGTCTTGATGCCCTTGGCAGCACCCTGCTAAGGAGGCAACAAGATG | |
